# The emergence of non-secretory multiple myeloma during the non-cytotoxic treatment of essential thrombocythemia: a case report

**DOI:** 10.1186/1752-1947-7-224

**Published:** 2013-09-11

**Authors:** Danijela Leković, Mirjana Gotić, Olivera Mitrović, Milica Radojković, Jelena Bila, Marija Dencic-Fekete, Nada Kraguljac-Kurtović, Maja Peruničić-Jovanović, Vladan Čokić

**Affiliations:** 1 Clinic for Hematology, Clinical Center of Serbia, 2 Koste Todorovica street, 11 000 Belgrade, Serbia; 2 Medical Faculty, University of Belgrade, Belgrade, Serbia; 3 Institute for Medical Research, University of Belgrade, Belgrade, Serbia; 4 Department of Hematology, Clinical Hospital Center “Dr Dragiša Misović”, Belgrade, Serbia

**Keywords:** Angiogenesis, Essential thrombocythemia, Multiple myeloma

## Abstract

**Introduction:**

The emergence of multiple myeloma as a second malignancy in patients with essential thrombocythemia is extremely rare. Several cases have been published so far, pointing out the impact of a cytotoxic effect during treatment of essential thrombocythemia on the development of multiple myeloma.

**Case presentation:**

We report the case of a 52-year-old Caucasian man who presented to our hospital because of leukocytosis, a slightly decreased hemoglobin level and thrombocytosis. After a complete hematological work-up, essential thrombocythemia was diagnosed. The patient was included in a multicenter clinical study, treated with anagrelide and his platelet counts were maintained in the normal range for more than 3 years. A sudden drop in his hemoglobin level with normal leukocyte and platelet count occurred at the same time as a back pain. Magnetic resonance imaging of his spine revealed the existence of a pathological fracture of Th4, the collapse of the upper edge of Th7 and osteolytic lesions of multiple thoracic vertebrae. Repeated hematological examinations, including bone biopsy with immunohistochemistry, disclosed diagnosis of multiple myeloma of the non-secretory type.

**Conclusions:**

To the best of our knowledge this is the first published case in which multiple myeloma developed during the treatment of essential thrombocythemia with the non-cytotoxic drug anagrelide. Our attempts to find a common origin for the coexistence of multiple myeloma and essential thrombocythemia have not confirmed the genetic basis of their appearance. Further studies are needed to determine the biological impact of this coexistence.

## Introduction

It is well known that essential thrombocythemia (ET) can progress to myelofibrosis or acute leukemia [1]. The occurrence of other secondary hematologic malignancies in patients with ET includes non-Hodgkin's lymphoma and myelodysplastic syndrome but development of multiple myeloma (MM) is extremely rare [2,3]. Several cases have been published so far, pointing out the impact of toxic effects during treatment of ET on the development of MM [4-6].

We report the case of a patient with ET who was treated with non-cytotoxic therapy anagrelide, in whom MM appeared almost 4 years after the diagnosis of ET. The expression of the angiogenic factors, vascular endothelial growth factor (VEGF), endothelial nitric oxide synthase (eNOS) and hypoxia-inducible factor 1-alpha (HIF1α), was analyzed in the bone marrow (BM) biopsy samples of our patient.

## Case presentation

**Figure 1:**
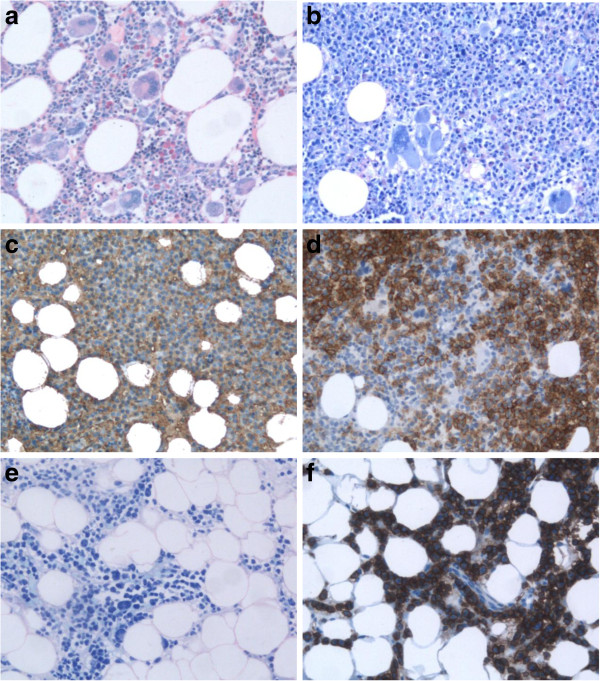
**Histological and immunohistochemical analysis of essential thrombocythemia and multiple myeloma. (a)** Initial bone marrow biopsy specimen when essential thrombocythemia was diagnosed (Giemsa, ×200). **(b)** Bone marrow biopsy specimen when multiple myeloma (MM) was diagnosed showing the diffuse infiltration with plasma cells (Giemsa, ×200). **(c)** Bone marrow biopsy specimen when MM was diagnosed showing the kappa positive plasma cells (×200). **(d)** Bone marrow biopsy specimen when MM was diagnosed showing the CD138 positive plasma cells (×200). **(e)** Bone marrow biopsy specimen after MM chemotherapy showing the diffuse infiltration with plasma cells (Giemsa, ×200). **(f)** Bone marrow biopsy specimen after MM chemotherapy showing the CD138 positive plasma cells (×200).

**Figure 2:**
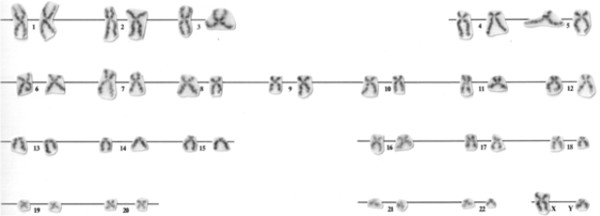
Karyogram from a bone marrow metaphase of the patient showing 46,XY.

A 52-year-old Caucasian man was referred to our clinic with leukocytosis, slightly decreased hemoglobin (Hb) level and thrombocytosis. He had dizziness in the last months. There was a history of 20 years of smoking and no exposure to any cytotoxic agents. His physical examination was normal. A complete blood cell count (CBC) showed: white blood cells (WBC)=14.1×10^9^/L with normal cell differentiation (61% of neutrophils, 7% of monocytes, 28% of lymphocytes, 3% of eosinophils and 1% of basophiles), Hb=135g/L, mean corpuscular volume 88fL, normal reticulocyte count and platelets (Plt)=1130×10^9^/L. Serum iron (9.6μmol/L, total iron-binding capacity=55.5μmol/L) and protein (serum total protein 77g/L, albumin 46g/L) analyses were normal. A chest X-ray, an ultrasound of his abdomen and an echocardiogram were normal. BM aspiration showed mainly sheets of megakaryocytes with normal marrow iron stores. A BM biopsy revealed megakaryocytic hyperplasia without signs of fibrosis, and large megakaryocytes with hyperlobated nuclei and a tendency to group in clusters (Figure 1a). Conventional cytogenetic testing showed a normal male karyotype 46,XY (Figure 2). Mutation of JAK2V617F was not detected in granulocytes of peripheral blood.

Based on these findings, the diagnosis of myeloproliferative neoplasm (MPN)-ET was made. The thromboreductive therapy with anagrelide within the multicenter clinical study was started when his Plt count was 1309×10^9^/L. The patient was treated for more than 3 years with a daily dose of 1mg. A complete hematological response was achieved after 1 month of treatment and thereafter Plt counts were maintained in normal values (from 176 to 423×10^9^/L) without any thrombotic or hemorrhagic complications.

**Figure 3:**
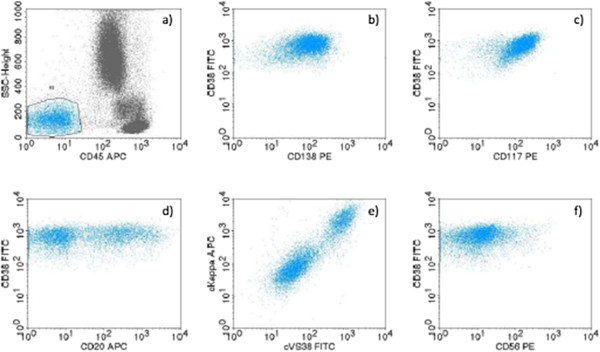
**Immunophenotypic profile of bone marrow multiple myeloma cells determined by flow cytometry. a)** CD45^-^, **b)** CD38^+high^, CD138^+high^, **c)** CD117^+high^, **d)** CD20^+hetero^, **e)** cyIgKappa^+hetero^, cVS38^+hetero^, **f)** CD56^-^ presented on biparametric histograms.

However, 3.5 years after ET diagnosis during a routine checkup, a drop in Hb level was observed (94g/L) with normal WBC (5.8×10^9^/L), and Plt (276×10^9^/L). Thromboreductive therapy was discontinued. Soon, the patient started to complain of persistent back pain. Magnetic resonance imaging of his spine revealed the pathological fracture of Th4, collapse of the upper limb of Th7 and multiple focal osteolytic lesions of thoracic vertebrae. A standard radiography showed multiple osteolytic lesions of his scalp, ribs, both humeri, then one osteolytic lesion of his left ischium, left proximal part of the radius and on the upper third of both femurs. In a CBC, a further decrease in his Hb level and Plt was observed (Hb=75g/L, Plt=33×10^9^/L). His erythrocyte sedimentation rate was 115mm/hour, C-reactive protein=12.8mg/L, urea=14mmol/L, creatinine=181μmol/L, calcium=3.5mmol/L, iron=46.6μmol/L, ferritin=1100ug/L, β-2 microglobulin=6.21mg/L and lactate dehydrogenase=1034U/L. His total serum protein (68g/L) and albumin (40g/L) were normal. His total urine protein level was 0.59g/L. His serum immunoglobulins (Ig) were slightly decreased: IgG=5.15g/L, IgA=0.64g/L, and IgM=0.19g/L. Immunofixation did not reveal any monoclonal protein in his serum and urine. Immunohistochemistry (IHC) of the BM biopsy revealed infiltration with 80% of monoclonal plasma cells (Figure 1b–d): LCA^-^, EMA^-^, Pax5, CD20^+/-^, CD3^-^, CD43^-^, CD38^+^, CD138^+^, kappa^+^, lambda^-^, IgG^+^, IgA^-^, IgM^-^, IgD^-^, CD31^-^, CD56^-^, Cyclin D1^-^, MUM1^+^, CD10^-^, and EBV^-^. Ki67^+^ was expressed in 20% of tumor cells (Figure 1c–f). Flow cytometry of the BM aspiration showed 8% of monoclonal plasma cells: CD38^+high^, CD138^+high^, CD117^+high^, cyVS38c^+hetero^, CD20^+hetero^, smIgKappa^+hetero^, cyIgKappa^+hetero^, CD19^-^, cCD79a^-^, CD45^-^, CD56^-^, CD52^-^, and CD10^-^ (Figure 3a–f). According to the above, the diagnosis of non-secretory MM, clinical stage IIIB (Durie–Salmon) was made, with the International Staging System – Stage 3. The patient was treated with cyclophosphamide, thalidomide, and dexamethasone (CTD) chemotherapy every 3 weeks (cyclophosphamide 500mg/kg/day D_1_, D_8_, D_15_; thalidomide 100mg/day every day; dexamethasone 40mg/day D_1–4_, D_12–15_). Zoledronic acid was administrated intravenously in doses of 4mg monthly for 6 months. Radiotherapy was applied on to Th4 for the pathological fracture and persistent back pain. After six courses of CTD, despite a very good performance status, the radiography revealed the presence of multiple osteolytic lesions and pathological fracture of Th4 and Th5. A BM biopsy with IHC showed persistent infiltration with 90% of monoclonal plasma cells. The patient continued treatment with vincristine, doxorubicin, and dexamethasone chemotherapy (vincristine 0.4mg/day D_1–4_; Adriamycin® (doxorubicin) 9mg/m^2^/day D_1–4_; dexamethasone 40mg/day D_1–4_, D_9–12_, D_17–20_) every 4 weeks, and received six courses without response. During palliative treatment with thalidomide 200mg/day, the patient died 22 months from MM diagnosis.

**Figure 4:**
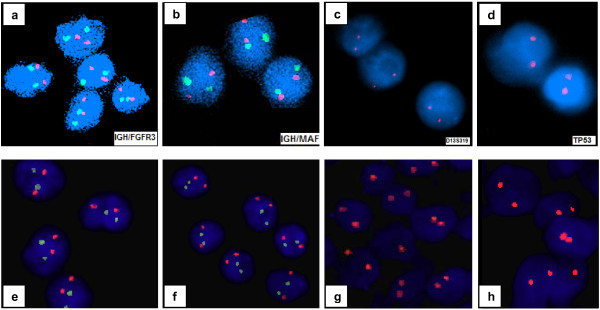
**Interphase fluorescence *in situ *hybridization analysis on paraffin-embedded bone marrow tissue at diagnosis of multiple myeloma and essential thrombocythemia.** Plasma cells nuclei negative for: **(a)** t(4;14)(p16;q32)- IGH/FGFR3 probe, **(b)** t(14;16)(q32;q23)- IGH/MAF probe, **(c)** del(13q14.3)-D13S319 probe, **(d)** del(17p13.1)- TP53 probe. Megakaryocytes nuclei negative for **(e)** t(4;14)(p16;q32)- IGH/FGFR3 probe, **(f)** t(14;16)(q32;q23)- IGH/MAF probe, **(g)** del(13q14.3)-D13S319 probe, **(h)** del(17p13.1)- TP53 probe.

Additional analyses were performed including interphase fluorescence *in situ* hybridization (FISH) on two paraffin-embedded BM biopsy specimens: the first specimen was from the MM diagnosis and the second from the ET diagnosis. FISH analyses for the t(4;14), t(14;16), del(17) and del(13) were done with Abbott-Vysis probes. In both paraffin specimens, a normal signal pattern was found in all analyzed nuclei (at least 100) for each applied FISH probe (Figure 4).

**Figure 5:**
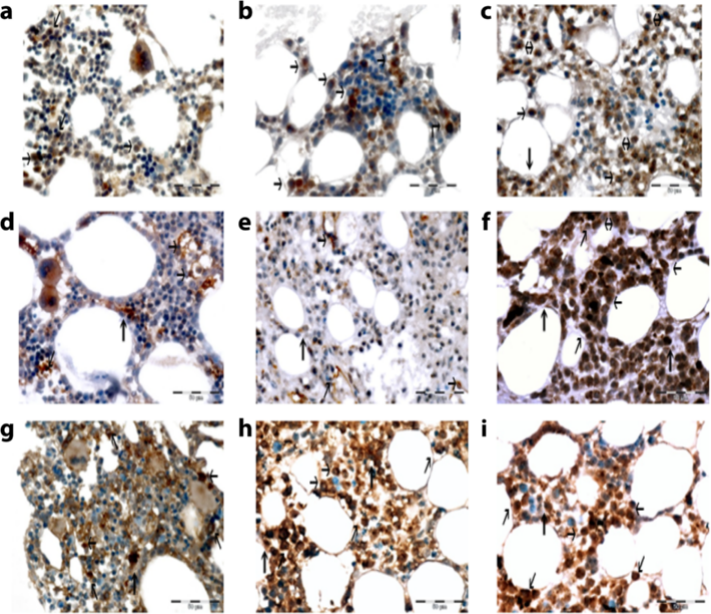
**Expression of vascular endothelial growth factor (VEGF), endothelial nitric oxide synthase (eNOS) and hypoxia-inducible factor 1-alpha (HIF1α) was analyzed using immunohistochemistry on bone marrow biopsy specimens of essential thrombocythemia (ET) and multiple myeloma (MM) (before and after chemotherapy).** VEGF expression: **(a)** at ET diagnosis, **(b)** at MM diagnosis, **(c)** in MM after chemotherapy. eNOS expression: **(d)** at ET diagnosis, **(e)** at MM diagnosis, **(f)** in MM after chemotherapy. HIF1α expression: **(g)** at ET diagnosis, **(h)** at MM diagnosis, **(i)** in MM after chemotherapy. Magnification x40. Immunoreactive cells are colored brown. The black arrows point toward positive cells.

The expression of angiogenic factors VEGF, eNOS and HIF1α was analyzed using IHC on BM biopsy specimens (Table 1). Immunoreactive complex was visualized with DAKO Liquid DAB+ Substrate-Chromogen System counterstained with Mayer's hematoxylin and evaluated under a light microscope. The results of the expression of angiogenic factors were obtained counting the number of positively stained endothelial cells using the hot spot method (Figure 5). Normal values of analyzed angiogenic factors were found at the time of ET diagnosis. However, VEGF and active nuclear form of HIF-1α were elevated at the time of MM diagnosis and all angiogenic factors were more than twice elevated after MM treatment.

**Table 1:** Percentage of vascular endothelial growth factor, endothelial nitric oxide synthase and hypoxia-inducible factor 1-alpha positive cells in essential thrombocythemia and multiple myeloma (before and after chemotherapy) on bone marrow biopsy samples of the patient compared to normal controls

**%**	**Vascular endothelial growth factor**	**Endothelial nitric oxide synthase**	**Hypoxia-inducible factor 1-alpha cytoplasmic/nuclear**
**Control**	**8**	**8.19**	**9.84 / 4.05**
**Essential thrombocythemia**	**7.39**	**8.08**	**8.15 / 0**
**Multiple myeloma**	**20.41**	**5.08**	**1.91 / 8.18**
**Multiple myeloma+chemotherapy**	**22.68**	**21.94**	**6.3 / 9.91**

## Discussion

ET is a MPN, characterized by the clonal proliferation of megakaryocytes in the BM and high Plt levels in peripheral blood [2]. By contrast, MM is a plasma cell neoplasm of post-germinal center, long-living plasma cells, in which the Ig genes have undergone a class switch with somatic hypermutation [7]. The non-secretory MM is a rare variant (1 to 5%) of the disease which is characterized by the clonal proliferation of the plasma cells in BM without detectable monoclonal protein in the serum or urine by conventional techniques [8]. The diagnosis often rests on the demonstration of monoclonal plasma cell infiltrates in the marrow as well as on the presence of lytic bone lesions, which are common in these patients. Myeloma plasma cells of the presented patient have shown typical immunophenotypic characteristics, detected by flow cytometry, but with two unusual findings: the first, concomitant expression of surface and cytoplasmatic form of the kappa light chain and CD20. These features are characteristics of early plasma cells and plasmablasts. The second unusual finding was the aberrant expression of CD117 and CD20 without the expression of the CD56 molecule. It was already observed that MM without CD56 expression might be associated with more aggressive disease and extramedullary dissemination [9].

According to previous reports, ET preceded MM in eight cases and both diseases were diagnosed simultaneously at their onset in three cases. MM had never preceded ET [4-6]. There is a report about a patient with ET who received busulphan plus hydroxyurea and then developed plasma cell leukemia [10].

The association of MM and ET is still unclear. Previous literature emphasizes the impact of cytotoxic effects in ET treatment on the development of second hematological malignancies [4-6]. One study based on the follow-up of 114 patients with ET, reported a higher incidence of second malignancies (leukemias, chronic lymphocytic leukemia and solid tumors) in hydroxyurea-treated patients (3.9%). Moreover, the significantly increased rate of second malignancies was found in a cohort of patients with ET sequentially treated with busulphan and hydroxyurea [11]. One of the largest studies, which involved the long-term follow-up of 331 patients with ET, observed second hematological malignancies in 15 patients (4.5%), including acute myeloid leukemia, acute lymphoblastic leukemia, non-Hodgkin lymphoma (follicular lymphoma and diffuse large B-cell lymphoma) and small lymphocytic lymphoma [3]. According to the type of ET treatment, second malignancies were documented in 7.3% of patients who did not receive any treatment, in 11.2% of those treated only with hydroxyurea, in 26.3% of those who received only alkylating agents, and in 25% of those treated with alkylating agents followed by hydroxyurea [3]. Considering the above findings, the occurrence of MM was not remarked upon during follow-up of patients with ET, indicating the sporadic coexistence of these two diseases.

Analyzing our clinical registry, in the last 30 years, among the 383 patients with MM, only this one was found to have MM in coexistence with ET. Concerning the treatment, our patient is unique because he was treated for ET with anagrelide, a non-cytotoxic therapy. Anagrelide is an efficient Plt-lowering agent in most patients with ET, including patients being treated for the first time and in those refractory to other thromboreductive therapy. Anagrelide exerts its effect by reducing the differentiation at the late stage of megakaryocytic development, which leads to reduced Plt production [12]. It has been observed in long-term follow-up that anagrelide-treated patients with ET did not develop acute leukemia or other hematological malignancies [12-14].

There are suggestions that MM clones have a higher proliferative potential compared with ET clones [5]. Nevertheless, the cytotoxic effect of anti-myeloma therapy on ET cannot be excluded. Previous publications have shown that angiogenesis could play an important role in the biology of hematological malignancies, including ET and MM. VEGF, a major angiogenic factor, was identified in higher concentrations in untreated patients with ET. However, in anagrelide-treated patients with ET, elevated VEGF and increased density of blood vessels were not observed [15,16]. By contrast, an increased level of angiogenesis is a regular characteristic of MM and has a prognostic value [17]. In our case, angiogenic factors were within normal ranges at the time of ET diagnosis. After the occurrence of MM, angiogenic factors increased despite treatment with the angiogenic drug thalidomide; that finding correlated with clinical resistance and continuous disease progression.

## Conclusions

To the best of our knowledge, this is the first published case in which MM developed during the treatment of ET with the non-cytotoxic drug anagrelide. Our attempts to find a common origin for the coexistence of MM and ET have not confirmed the genetic basis of their appearance. Further studies are needed to determine the biological impact of this coexistence.

## Consent

Written informed consent was obtained from the patient for publication of this case report and any accompanying images. A copy of the written consent is available for review by the Editor-in-Chief of this journal.

## Abbreviations

BM: Bone marrow; CBC: Complete blood cell count; CTD: Cyclophosphamide, thalidomide, and dexamethasone; eNOS: Endothelial nitric oxide synthase; ET: Essential thrombocythemia; FISH: Fluorescence *in situ* hybridization; Hb: Hemoglobin, HIF1α, Hypoxia-inducible factor 1-alpha; Ig: Immunoglobulin; IHC: Immunohistochemistry; MM: Multiple myeloma; MPN: Myeloproliferative neoplasm; Plt: Platelet; VEGF: Vascular endothelial growth factor; WBC: White blood cells.

## Competing interests

The authors declare that they have no competing interests.

## Authors’ contributions

DL and MG were responsible for the patient diagnosis and follow-up, interpreted the results and wrote the paper; MDF did the FISH analyses; MPJ and OM did the IHC analyses; VC did the angiogenic analyses; JB and MR took care of the patient during chemotherapy; NKK did the immunophenotypic analyses. All authors read and approved the final manuscript.
